# Strong Prediction: Language Model Surprisal Explains Multiple N400 Effects

**DOI:** 10.1162/nol_a_00105

**Published:** 2024-04-01

**Authors:** James A. Michaelov, Megan D. Bardolph, Cyma K. Van Petten, Benjamin K. Bergen, Seana Coulson

**Affiliations:** Department of Cognitive Science, University of California, San Diego, La Jolla, CA, USA; Department of Psychology, Binghamton University, State University of New York, Binghamton, NY, USA

**Keywords:** distributional semantics, ERPs, N400, neural language models, predictive coding

## Abstract

Theoretical accounts of the N400 are divided as to whether the amplitude of the N400 response to a stimulus reflects the extent to which the stimulus was predicted, the extent to which the stimulus is semantically similar to its preceding context, or both. We use state-of-the-art machine learning tools to investigate which of these three accounts is best supported by the evidence. GPT-3, a neural language model trained to compute the conditional probability of any word based on the words that precede it, was used to operationalize contextual predictability. In particular, we used an information-theoretic construct known as surprisal (the negative logarithm of the conditional probability). Contextual semantic similarity was operationalized by using two high-quality co-occurrence-derived vector-based meaning representations for words: GloVe and fastText. The cosine between the vector representation of the sentence frame and final word was used to derive contextual cosine similarity estimates. A series of regression models were constructed, where these variables, along with cloze probability and plausibility ratings, were used to predict single trial N400 amplitudes recorded from healthy adults as they read sentences whose final word varied in its predictability, plausibility, and semantic relationship to the likeliest sentence completion. Statistical model comparison indicated GPT-3 surprisal provided the best account of N400 amplitude and suggested that apparently disparate N400 effects of expectancy, plausibility, and contextual semantic similarity can be reduced to variation in the predictability of words. The results are argued to support predictive coding in the human language network.

## INTRODUCTION

Fedorenko and Thompson-Schill (2014) note that the brain systems that support language processing are better described at the level of interactive networks than individual brain regions, arguing that investigations into the functional significance of neural activity are best directed at large-scale distributed neural networks, that is, a set of interconnected brain regions acting in concert. This may explain why language researchers have found event-related brain potentials (ERPs) to be such a useful method for probing the neurobiology of language, despite known limitations in the spatial resolution of the technique (see Federmeier et al., 2016, for a review). Electroencephalography (EEG) reflects post-synaptic potentials generated mainly in cortical pyramidal cells (Luck, 2014). Moreover, brain activity cannot be detected at the scalp unless large numbers (on the order of 10 million) of neurons are simultaneously active (Woodman, 2010). The identification of any scalp recorded potentials whose amplitude is systematically modulated by language processing demands is thus likely to reflect activity in the very sort of interactive neural networks Fedorenko and Thompson-Schill (2014) propose.

One ERP component of particular interest to language researchers is the N400, a monophasic negativity peaking approximately 400 ms after the onset of a visually presented word. The N400 was first reported in a study that compared ERPs elicited by the last word of sentences that made sense (*He takes his coffee with cream and **sugar***) versus those that did not (*He takes his coffee with cream and **dog***; Kutas & Hillyard, 1980). However, it soon became clear that the N400 is not observed only at the end of sentences; it is elicited by all words, written, spoken, or signed, and that its amplitude is modulated by factors such as contextual congruity, frequency of usage, and category membership, all thought to affect the difficulty of retrieving information in semantic memory (for review, see Kutas & Federmeier, 2011).

Here we consider the adequacy of two proposals regarding the functional significance of the N400 that differ in their implications for the underlying neurocognitive mechanisms. The first is that N400 amplitude is sensitive to the conditional probability of words in their linguistic contexts as driven by a predictive coding mechanism. This account is referred to below as *predictive preactivation*. The second is that N400 amplitude is driven by a context-sensitive retrieval mechanism and as such indexes the semantic similarity of incoming words to the semantic features of prior words in the context. This is referred to below as *contextual semantic similarity*. We briefly review empirical support for each of these proposals as well as that for a combined account.

One reason for the continued dispute on this issue is that advocates of each account have mostly focused on a subset of N400 effects, discounting the relevance of less amenable phenomena and arguing that they are potentially explicable given a suitable operationalization of either expectancy or semantic similarity. Whereas advocates of predictive processing focus on expectancy effects (Bornkessel-Schlesewsky & Schlesewsky, 2019; DeLong, Troyer, & Kutas, 2014; Kuperberg et al., 2020; Kuperberg & Jaeger, 2016), advocates of contextual similarity and combined accounts focus on the way that N400 amplitude is modulated by the presence of semantically related words in the immediate context (Ettinger et al., 2016; Federmeier, 2022; Lau et al., 2013; Uchida et al., 2021). By contrast, the present study examines manipulations of the expectancy, plausibility, and relatedness of sentence final words to the words that precede them.

Noting how researchers in the neurobiology of language have struggled to operationalize the theoretical constructs proposed to drive the N400, we turn instead to tools from computational linguistics. The 21st century has seen immense progress in the utility of statistical tools designed to characterize the features and probabilities of words in texts (Berger & Packard, 2022; Jurafsky & Martin, 2023). Trained on large corpora, such tools are used in applications such as information retrieval, speech recognition, machine translation, and chatbots. Although they are not proposed as cognitive models per se, we suggest that the data-driven estimates they provide serve as excellent metrics for the theoretical constructs proposed to drive the N400. We utilize three state-of-the-art natural language technologies—one language model and two sets of word vectors—to provide metrics for the predictability and the contextual semantic similarity (respectively) of our sentence-final words and compare their adequacy in accounting for N400 effects of expectancy, plausibility, and relatedness in human participants.

### Predictive Preactivation Account

One prominent account of the N400 is that it reflects the activation of semantic features associated with the eliciting word (Kutas & Federmeier, 2011). According to this account, contextual congruity effects occur because elements of the prior context have already activated some of these associated features. If relevant features associated with a word have been activated by the preceding context—whether these be semantic features (Federmeier, 2022; Kuperberg et al., 2020) or a combination of semantic, grammatical, and phonological features (as supported by the work of DeLong et al., 2005; Fleur et al., 2020; Nicenboim et al., 2020; Otten et al., 2007; Urbach et al., 2020; Van Berkum et al., 2005)—they need not be newly activated when the word is encountered, and thus the amplitude of the N400 is less than when words are encountered alone or in less supportive contexts.

The most obvious source of support for predictive preactivation lies in the close relationship between N400 amplitude and the expectancy metric known as *cloze probability* (the proportion of people to fill in the relevant gap in a sentence with a given word; Taylor, 1953, 1957). A higher-cloze continuation of a sentence elicits a smaller (i.e., more positive) N400 response, while a lower-cloze continuation elicits a larger (more negative) N400 (Kutas & Federmeier, 2011; Kutas & Hillyard, 1984). In fact, in previous work the two variables have been reported to have a Pearson correlation coefficient *r* of −0.9 or more (Kutas & Federmeier, 2011; Kutas & Van Petten, 1994). As the cloze task requires participants to predict an upcoming word, cloze probability has often been argued to reflect how predictable a word is in context (Brothers & Kuperberg, 2021; Fischler & Bloom, 1979; Kuperberg et al., 2020; Kutas et al., 2011; Kutas & Hillyard, 1984; Luke & Christianson, 2016; Tannenbaum et al., 1965; Van Petten & Luka, 2012). Moreover, the negative correlation between N400 amplitude and cloze probability tells us that N400 amplitude is not simply a categorical indicator of surprise, but reflects the predictability of the eliciting word in a more fine-grained way.

Beyond the graded predictability effect, the predictive preactivation account is supported by the way in which N400 amplitude is modulated by sentence context. Research has shown that words elicit a large N400 when presented alone, a large N400 when presented in sentence frames that render them unexpected, and a progressively smaller N400 in more supportive sentence contexts, suggesting that what reduces the amplitude of the response is the activation of neural representations associated with the stimulus before the stimulus is encountered (Dambacher et al., 2006; Payne et al., 2015; Van Petten, 1993; Van Petten & Kutas, 1990, 1991; for discussion, see Federmeier, 2022; Van Petten & Luka, 2012). Second, unlikely sentence continuations elicit a similar-sized N400 in constraining contexts in which there is a highly salient alternative (e.g., **month** in *The bill was due at the end of the **hour***) and in open-ended contexts in which there is not (e.g., *He kicked himself when he realized that he forgot the **key***; see DeLong & Kutas, 2020; Federmeier, 2022; Kuperberg et al., 2020; Van Petten & Luka, 2012).

This sensitivity to the contextual fit of the actual word encountered rather than the predictability of potential alternatives has been interpreted as suggesting that rather than the registration of surprise, the N400 reflects the activation of semantic (and possibly other) features associated with the word presented. Under this account, cloze probability effects occur because the greater the extent of preactivation for a word’s features, the smaller the N400 elicited by the word (DeLong & Kutas, 2020; DeLong, Quante, & Kutas, 2014; Federmeier, 2022; Kuperberg et al., 2020; Kutas et al., 2011; Kutas & Federmeier, 2011; Van Petten & Luka, 2012).

In addition to cloze, the amplitude of the N400 is also correlated with other metrics of predictability. Research has found that the predictions of language models, computational systems designed to predict the probability of a word in context based on the statistics of language, are correlated with the N400 response to these words (Aurnhammer & Frank, 2019; Frank et al., 2015; Merkx & Frank, 2021). Specifically, such studies find that surprisal, the negative logarithm of the conditional probability of a word, is a significant predictor of N400 amplitude (Aurnhammer & Frank, 2019; Ettinger, 2020; Frank et al., 2015; Merkx & Frank, 2021; Michaelov & Bergen, 2020; Michaelov et al., 2023; Parviz et al., 2011; Szewczyk & Federmeier, 2022).

Research also shows that language model surprisal can be used to model N400 effects—in many cases, where we find a significant difference in N400 amplitude between stimuli from two experimental conditions, we also find a significant difference in surprisal in the same direction (Michaelov & Bergen, 2020). Further, this computational approach fits into a larger body of work showing that N400 amplitude is sensitive to the statistics of language—for example, more frequent words elicit smaller N400 responses (Dambacher et al., 2006; Fischer-Baum et al., 2014; Kutas & Federmeier, 2011; Rugg, 1990; Van Petten, 1993; Van Petten & Kutas, 1990). These results together suggest that the N400 component reflects a neural process that veridically tracks the conditional probability of upcoming words. Note that the definition of conditional probability here is not restricted to that calculated by a traditional n-gram model, only based on actual co-occurrences of lexical items; language models are designed to generalize based on their training data when making predictions, and humans are also thought to do so (DeLong & Kutas, 2020; DeLong, Troyer, & Kutas, 2014; Kuperberg et al., 2020).

### Contextual Semantic Similarity

An alternative explanation of the neural activity underlying the N400 is contextual semantic similarity. Under this account, as we comprehend a sentence, the semantic features of each word are activated and briefly maintained, thereby reducing the neural activity required in response to words with overlapping features (Federmeier, 2022). While this feature-based account is compatible and indeed central to some prediction-based accounts of the N400 (e.g., Kuperberg et al., 2020), the key difference is that the activations are limited to semantic features of previously encountered words. That is, there is no additional spreading activation to related words or semantic features, and, crucially, no prediction. Some investigators have suggested that contextual semantic similarity may be able to account for all variation in N400 amplitude (Ettinger et al., 2016; Uchida et al., 2021), while others suggest semantic similarity acts in concert with a prediction mechanism (see, e.g., Federmeier, 2022; Frank & Willems, 2017; Lau et al., 2013).

Several previous ERP studies have examined the impact of semantically related words within sentences or sentence-like word strings, with results that suggest the N400 component is sensitive to semantic similarity among the individual words that comprise sentences along with factors that are difficult to accommodate within a pure similarity account. For instance, an early experiment found that the relationship between the two terms of a statement about category membership influenced the N400, whereas the truth or falsity of the statement had no impact, so that *a robin is a **bird*** and *a robin is not a **bird*** were equivalent and both led to smaller N400s than *a robin is*/*is not a **vehicle*** (Fischler et al., 1984). Similarly, Kounios and Holcomb (1992) found no impact of quantifiers *all*, *some*, and *no* on statements about category membership. However, a more recent study on this topic reports N400 effects both for relationships between words (viz., *farmers* primes *crops* more than *farmers* primes *worms*) as well as a small N400 effect of quantifiers, that is, the final word of the more plausible sentence *farmers often grow **crops*** elicited a smaller N400 than *farmers rarely grow **crops*** (Urbach & Kutas, 2010).

Outside the realm of negation and quantification, initial studies showed that the presence of a strongly related word within either a meaningful sentence (e.g., *When the **moon** is full, it is hard to see many **stars** or the Milky Way*) or a grammatically legal but meaningless word string (e.g., *When the **moon** is rusted, it is available to buy many **stars** or the Santa Ana*) leads to a smaller N400 to **stars** than if the prior context does not include a related word (Van Petten, 1993; Van Petten et al., 1997). However, other studies indicate that N400 is not driven solely by an automatic semantic comparison process during sentence comprehension. Coulson and colleagues found much smaller N400s to the second words of related (*tin*/***aluminum***) than unrelated (*tin*/***disposal***) word pairs when the pairs were presented by themselves (Coulson et al., 2005). The word pairs were then embedded in sentences that were compatible or incompatible with the word-pair relationship, like the quartet below.

(1)  (a) Coke cans used to be made out of tin but now they use **aluminum**.
    (b) Paul heard a loud grinding noise when someone put a tin can right down the garbage **aluminum**.
    (c) Paul heard a loud grinding noise when someone put a tin can right down the garbage **disposal**.
    (d) Coke cans used to be made out of tin but now they use **disposal**.

In the incongruous sentences, the presence of a semantically related word continued to reduce the amplitude of the N400 elicited by the final words—condition (b) smaller than (d)—but this difference was dramatically smaller and shorter in duration than when the word pairs were presented in isolation. In contrast, the impact of overall sentence congruity—conditions (a) and (c) versus (b) and (d)—dwarfed the impact of a single related word earlier in the sentence.

Camblin et al. (2007) similarly pitted overall plausibility against lexical relationships by embedding strongly related word pairs (*arms*/*legs*) in discourse contexts that were more or less compatible with the word-pair relation (skin irritation from a sunburn would be likely to affect both arms and legs, but irritation from a wool sweater would not). Much like Coulson et al. (2005), they found smaller N400s for the second words of semantically similar pairs than their unrelated controls, but that this effect was substantially smaller when opposed by the global discourse context.

As is the case for the prediction account, the contextual semantic similarity account is supported by work with computational models. N400 amplitude, for example, has been found to correlate with the degree of semantic similarity between prime and target word (Chwilla & Kolk, 2005; Van Petten, 2014), as operationalized by latent semantic analysis (LSA), a measure of semantic distance derived from word co-occurrence frequencies in written corpora (Dumais, 2004; Dumais et al., 1988; Landauer et al., 1998). This is also true for words in sentence contexts—N400 amplitude is correlated with the LSA distance between a target word and the words that precede it (Chwilla et al., 2007; Parviz et al., 2011), and with other statistically derived metrics of word similarity (Broderick et al., 2018; Ettinger et al., 2016; Frank & Willems, 2017; Parviz et al., 2011; Uchida et al., 2021; Van Petten, 2014).

### Multiple Systems Accounts

A number of investigators have suggested the brain activity underlying the N400 reflects both predictive preactivation and contextual semantic similarity. Some of these suggest that the contextual semantic similarity system operates by default, and the predictive system is engaged under conditions of increased attention (Federmeier, 2022), or when predictions are more likely to be successful, as when a high proportion of word pairs are semantically related (Holcomb, 1988; Lau et al., 2013). Some studies have shown that conditions that foster prediction result in N400 effects with an earlier onset latency than conditions that do not, such as those with little time between words (Anderson & Holcomb, 1995; Luka & Van Petten, 2014) or a small proportion of related word pairs (Lau et al., 2013).

According to other accounts, both systems are constantly active but implemented in different brain circuits. In one functional magnetic resonance imaging (fMRI) experiment, Frank and Willems (2017) found that contextual semantic similarity was correlated with activations in the anterior middle temporal sulcus, the precuneus, and bilateral angular gyri, whereas predictability was correlated with activations in the left inferior temporal sulcus, left posterior fusiform gyri, bilateral superior temporal gyri, and bilateral amygdalae. In view of the limited temporal resolution of fMRI, however, it is also possible that these findings reflect a disparate impact of contextual similarity and predictability at distinct stages of language processing.

Finally, one well-replicated result appears challenging to accommodate in single-system accounts, whether predictive or similarity based. Kutas and Hillyard (1984) first reported that generally poor (unexpected) sentence completions elicited smaller N400s if they were semantically related to the most expected completion than if not, so that *He liked lemon and sugar in his **coffee*** led to a less negative ERP than an equally unexpected word (***dog***) that is semantically dissimilar to the expected completion (***tea***). The finding that words related to the best completion elicit significantly less negative N400 responses than their unrelated counterparts has been replicated many times, and occurs regardless of whether the related words comprise congruous or anomalous continuations of a sentence (Amsel et al., 2015; DeLong et al., 2019; Federmeier & Kutas, 1999; Ito et al., 2016; Kutas, 1993; Kutas et al., 1984; Kutas & Hillyard, 1984; Thornhill & Van Petten, 2012). One might imagine that this effect (relationship-to-best-completion, or RBC) arises from predicting a sentence completion, followed by an assessment of the similarity between that prediction and the actually delivered word, but no study has suggested that the RBC effect is temporally delayed relative to simple sentence congruity effects. Because an RBC condition is included in the present study, we return to theoretical accounts and attempts to computationally model it in the Discussion.

### The Present Study

In the present study we explore whether the brain activity underlying the scalp-recorded N400 component is driven by predictability, contextual semantic similarity, or a combination of the two. To do so, we recorded EEG as participants read sentences whose final words were designed to elicit three kinds of N400 effects: predictability, plausibility, and RBC. Based on the stimuli used by Thornhill and Van Petten (2012), our materials were sentence frames with four different kinds of sentence-final words. As in the original study, the predictability manipulation was guided by results from a cloze task. The Best Completion condition was thus the word with the highest cloze probability. The Related completions were low-cloze completions semantically related to the best completions, as determined by Thornhill and Van Petten (2012). Likewise the Unrelated completions were low-cloze completions unrelated to the best completions. Finally, to investigate the plausibility effect, we included Implausible completions, completions with a cloze probability of zero that were also implausible.

(2)  (a) Best Completion: On his vacation, he got some much needed **rest**.
    (b) Related: On his vacation, he got some much needed **relaxation**.
    (c) Unrelated: On his vacation, he got some much needed **sun**.
    (d) Implausible: On his vacation, he got some much needed **airlines**.

We then use state-of-the-art language models to calculate the predictability and contextual similarity of our stimuli and investigate how well these metrics predict the single-trial N400 amplitudes elicited by the stimuli. To operationalize predictability, we used the transformer neural network language model, GPT-3. Research has shown that in general, larger language models trained on more data provide the best fits to human data, and that transformer neural networks are the architecture best suited to predicting N400 data (Merkx & Frank, 2021). However, rather than using the conditional probabilities assigned by GPT-3 to our stimuli, we instead utilize surprisal scores, the negative logarithm of the probability assigned by the language model to a given word in context. Previous work has shown that when directly compared, language model surprisal is a better predictor of N400 amplitude than raw probability (Szewczyk & Federmeier, 2022; Yan & Jaeger, 2020).

Contextual semantic similarity is generally calculated as the cosine distance between a vector representation of the stimulus word (often referred to as an embedding) and the mean vector across each word in the context, where the vector representations are based on the statistics of language. To operationalize contextual semantic similarity, we took advantage of two different tools for obtaining vectors for word meanings, GloVe (Pennington et al., 2014) and fastText (Bojanowski et al., 2016; Mikolov et al., 2018). GloVe (Pennington et al., 2014) is an unsupervised learning algorithm trained on global, aggregated word–word co-occurrence statistics that yields vector representations for words. The fastText library (Bojanowski et al., 2016) is an updated version of word2vec (Mikolov, Chen, et al., 2013; Mikolov, Sutskever, et al., 2013), which has been used in previous work investigating the effect of contextual semantic similarity (Ettinger et al., 2016; see also Frank & Willems, 2017, and Nieuwland et al., 2020, for related approaches). Both models are driven by language statistics, but GloVe embeddings are derived from co-occurrence statistics from a whole corpus (Pennington et al., 2014), while fastText embeddings are retrieved from a neural network (known as a continuous bag-of-words model) trained to predict a word based on the other words occurring in a given sentence (Bojanowski et al., 2016; Mikolov et al., 2018).

We expect that our experimental manipulation of predictability, plausibility, and RBC will replicate each of these well-documented effects on the N400, as would be evidenced by an effect of experimental condition. In particular, we expect the Best completions to elicit the least negative (most positive) N400, the Implausible completions to elicit the most negative N400, and the Related and Unrelated completions to fall in between the two. Despite the fact that the Related and Unrelated completions are matched for cloze probability and plausibility, the Related completions are expected to elicit smaller N400 than Unrelated completions.

Next we use our metrics of predictability and contextual semantic similarity to model single-trial N400 data using linear mixed effects regressions. If the brain activity underlying the N400 reflects predictive preactivation, we expect regressions incorporating surprisal to provide the best account of the data. Alternatively, if the brain activity underlying the N400 reflects contextual semantic similarity, we expect regressions incorporating one of our cosine similarity measures to provide the best account of the data. Finally, if the N400 reflects the operation of both a predictive preactivation mechanism and contextual similarity mechanism, the best account of the data will lie in regressions that incorporate measures both for surprisal and cosine similarity.

## MATERIALS AND METHODS

### Participants

Fifty University of California, San Diego (UCSD) volunteers participated for course credit or payment. Participants were right-handed, fluent English speakers with normal or corrected-to-normal vision with no history of neurological or psychiatric disorders. Participants ranged in age from 18 to 31 years old.

### Materials

Our stimuli were based on the original stimuli of the experiment carried out by Thornhill and Van Petten (2012). These stimuli were of the form given in Table 1. For each sentence frame, the stimuli from the Thornhill and Van Petten (2012) study fall under three conditions—Best Completions, the completions with the highest cloze probability; Related Completions, low-cloze completions that are semantically related to the best completions (as determined by Thornhill & Van Petten, 2012); and Unrelated Completions, low-cloze completions that are unrelated to the best completions. Thornhill and Van Petten (2012) found that these stimuli elicit both a predictability and RBC effect in human comprehenders. In order to also investigate the plausibility effect, we added a fourth experimental condition of Implausible Completions.

**Table 1.** Descriptive statistics for sentences: mean and standard deviation (*SD*) of cloze probabilities and plausibility ratings (1 = *very plausible*; 5 = *very implausible*) for each experimental condition.

Condition	Example stimulus	Cloze		Plausibility	
		Mean	*SD*	Mean	*SD*
Best	*It’s hard to admit when one is **wrong***.	49.8%	27.3%	1.4	0.3
Related	*It’s hard to admit when one is **incorrect***.	2.3%	3.3%	1.5	0.4
Unrelated	*It’s hard to admit when one is **lonely***.	2.3%	3.9%	1.5	0.3
Implausible	*It’s hard to admit when one is **screened***.	0%	0%	4.3	0.4

Sentences were normed via online surveys using the same participant pool we used to recruit participants for the EEG study. First, cloze probability measures were collected from UCSD students such that each sentence frame was completed by at least 35 participants. In this survey, participants were provided with sentence frames and instructed to produce the last word of the sentence. Average cloze probability and standard deviation for each condition are shown in Table 1.

All sentences were also rated for plausibility by a separate group of at least 30 students. In this survey, participants read one sentence at a time and were asked to rate each on a scale from 1 (*very plausible*) to 5 (*very implausible*). Multiple stimulus lists were employed so that each participant viewed only one of the four versions of each sentence frame. Average plausibility ratings for each experimental condition are shown in Table 1. All sentences in the Implausible condition had ratings above 3.5, with an average rating of 4.3. By contrast, the other conditions all had ratings below 2, suggesting participants found them plausible.

These stimuli were initially constructed as part of a larger study. In order to use the computational tools required to test our hypotheses, we opted to analyze a subset of the data such that critical words of all sentence stimuli appeared as whole tokens in GPT-3, GloVe, and fastText—that is, critical words were present as whole words in the vocabularies of these models. We then further selected stimuli such that, as in Thornhill and Van Petten (2012), there was no overall difference in cloze probability between the related and unrelated completions. We also additionally ensured that there was no overall difference in plausibility. Thus, the two conditions differed only in how related they were to the Best Completion for that sentence. This resulted in a final stimulus set of 125 sentence frames in four conditions, for a total of 500 items. The stimuli were presented along with 165 other sentences that were part of the larger study and thus similar in character to the experimental sentences. As for the experimental sentences, these additional stimuli were equally likely to end with the Best, Related, Unrelated, or Implausible completion for the sentence frame as each participant saw approximately 41 non-experimental stimuli in each condition—in addition to the approximately 31 experimental sentences in each condition.

### Procedure

Testing consisted of a single experiment session, with words presented centrally using RSVP presentation. For each sentence, participants first saw a break screen, then pressed a key to display the sentence. A fixation character remained on the screen while words were presented for 300 ms, followed by a 200 ms blank screen. The final word was displayed for 1,200 ms. After some sentences, participants saw a question about the content of the previous sentence (e.g., “Was the previous sentence about banking?”) and responded Yes or No with a button press.

### EEG Recording and Analysis

The EEG was recorded from 29 electrodes in an Electro-cap organized in the International 10–20 configuration. Additional electrodes were placed below the eye and near the external canthi to detect eye movements and blinks. Scalp electrodes were referenced online to an electrode on the left mastoid, and later re-referenced to an average of the left and right mastoid electrodes. The EEG was amplified using an SA Instrumentation bioelectric amplifier, digitized online at 250 Hz.

EEG was time locked to the onset of each sentence final word. Mean voltage during the 100 ms interval preceding each word’s appearance was used to baseline epochs spanning 100 ms before until 900 ms after word onset. Trials containing artifacts due to blinks, eye movements, or amplifier saturation were removed prior to analysis. As discussed in Materials, the data used in the present study were collected as part of a larger experiment involving additional stimuli constructed to cover the same four conditions. We analyzed all the data for stimuli that fulfilled the requirements stated in Materials, namely, stimuli where all critical words existed as whole words in all language models’ and word embeddings models’ vocabularies and Related and Unrelated words were matched for Cloze and Plausibility.

N400 amplitude was operationalized as the mean voltage 300–500 ms post-onset recorded from nine centroparietal electrodes: C3, Cz, C4, CP3, CPz, CP4, P3, Pz, and P4. All graphs and statistical analyses were run in R (R Core Team, 2022) using RStudio (RStudio Team, 2020) and the tidyverse (Wickham et al., 2019), lme4 (Bates et al., 2015), lmerTest (Kuznetsova et al., 2017), corrr (Kuhn et al., 2022), colorspace (Zeileis et al., 2009, 2020), gridExtra (Auguie, 2017), and cowplot (Wilke, 2020) packages. All figures use colorblind-friendly palettes (Chang, 2022; Jackson, 2016; Zeileis et al., 2020). All reported *p* values are corrected for multiple comparisons based on false discovery rate (Benjamini & Yekutieli, 2001).

### Computational Metrics

In this article, we derive three computational metrics based on the statistics of language—GPT-3 surprisal, GloVe cosine similarity, and fastText cosine similarity. While the pretrained models we used differ in a number of ways, we did attempt to match some of their properties as much as possible. For example, GPT-3, GloVe, and fastText are all trained on Common Crawl data (https://commoncrawl.org/), albeit using subsets of different sizes. GPT-3 is trained on 300 billion tokens, GloVe on 840 billion, and fastText on 600 billion tokens. In spite of these differences, at a minimum the corpus is the same and the training set is the same order of magnitude for all three models. Further, to ensure that all the models are equally able to capture the relationships between the stimuli and their contexts, stimuli were chosen such that critical words existed as whole words in all models’ vocabularies. For this reason, we use the version of fastText that does not include subword information in its representations, as the other models do not have access to subword information. More details on how each metric was calculated are provided below.

#### GPT-3 surprisal

The OpenAI API (OpenAI, 2021) was used to access the predictions of the largest original GPT-3 model (*Davinci*), which has 175 billion parameters (Brown et al., 2020). Each sentence stimulus was input into the API, and GPT-3 was used to calculate the log probability of the final word given its preceding context. Since these log probabilities used the natural exponent as a base, they were converted to the logarithm of base two and multiplied by negative one. The resultant surprisal values are thus measured in bits (see, e.g., Futrell et al., 2019, for discussion).

#### GloVe cosine similarity

To obtain the measure of contextual similarity we refer to as GloVe contextual cosine similarity, we used the GloVe (Pennington et al., 2014) vectors made available through the GloVe website (https://nlp.stanford.edu/projects/glove/)—specifically, the version with a 2.2 million word vocabulary and 300-dimensional vectors trained on 840 billion tokens from the Common Crawl corpus. We took the mean vector of all the words preceding the stimulus word and then used SciPy (Virtanen et al., 2020) to calculate the cosine similarity between this vector and the vector corresponding to the stimulus word. Because cosine similarity is based on the angle between two vectors and is not affected by the overall magnitude, this approach is equivalent to taking the sum of the context vectors as in Frank and Willems (2017).

We also calculate the similarity between the best completion (i.e., highest-cloze sentence completions) and each critical word in each sentence frame, which we refer to as GloVe best completion cosine similarity or GloVe BCCS.

#### fastText cosine similarity

To calculate fastText contextual cosine similarity, we utilized the fastText (Bojanowski et al., 2016) vectors made available through the fastText website (https://fasttext.cc/)—specifically, the version with a 2 million word vocabulary, 300-dimensional vectors, and no subword information trained on 600 billion tokens from the Common Crawl corpus. As with the GloVe vectors, we calculated the cosine similarity between the vector corresponding to the stimulus word and the mean vector of the preceding context. In addition to calculating fastText contextual cosine similarity (fastText CCS), we also calculate fastText best completion cosine similarity (fastText BCCS).

## RESULTS

**Figure 1.**
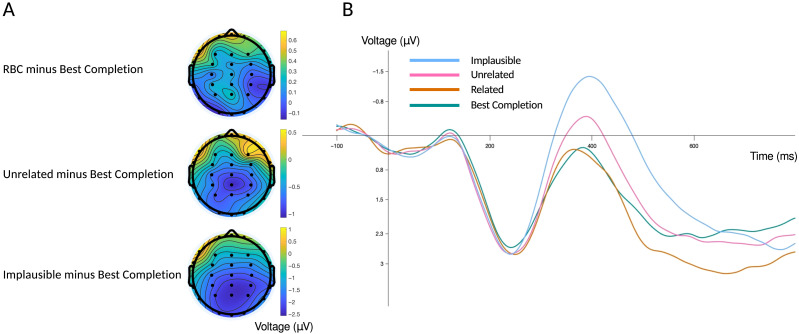
Event-related potential (ERP) scalp maps and waveforms. (A) Topography of the mean amplitude 300–500 ms of the difference wave for the Relatedness-to-Best-Completion (RBC) and Best Completion conditions (top), Unrelated and Best Completion (middle), and Implausible and Best Completion (bottom) using a spherical spline interpolation. (B) ERP waveforms for each condition (Best Completion, Related, Unrelated, Implausible) as measured at the centroparietal electrode cluster used in the regression models.

Figure 1 shows grand average ERP waveforms for words in each of the four conditions (Best Completion, Related, Unrelated, and Implausible) along with topographic maps. By convention, negative voltage is plotted upwards making it apparent that, as predicted, the Implausible condition elicited the largest (most negative) N400, and the Best Completions elicited the smallest (most positive) N400. The Unrelated condition fell in between these two extremes, and, as predicted, elicited more negative ERPs than did the Related condition (which was virtually overlapping the Best Completion condition, despite the large difference in their average cloze probability). The topographic maps were formed by first calculating point-by-point difference waves obtained by subtracting the amplitude of ERPs recorded at each electrode in the Best Completion condition from their counterparts in the Related, Unrelated, and Implausible conditions, respectively. The mean amplitude 300–500 ms was then measured on each difference wave and plotted on the scalp to visualize the relative pattern of positive and negative voltage. The posterior negativity apparent in all three plots is characteristic of N400 ERP effects reported in sentence reading paradigms like the one used here.

**Figure 2.**
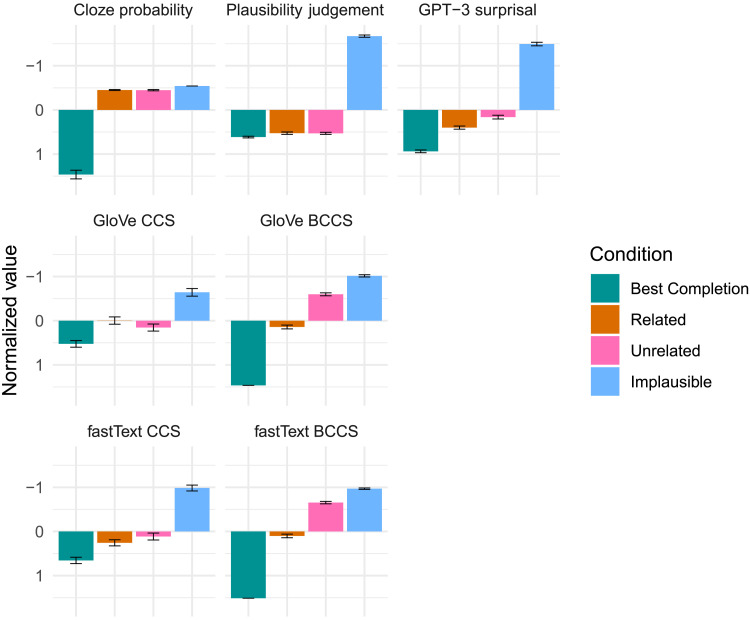
Average values of all predictors under each experimental condition. For easier comparison across predictors, we plot negative surprisal and plausibility, and the values of all predictors were *z*-scored. For easier comparison to the N400 waveform, the *y*-axis is reversed, with negative values plotted upwards. Error bars show the standard error. BCCS = best completion cosine similarity; CCS = contextual cosine similarity.

Figure 2 presents normalized (*z*-scored; and in the case of surprisal and plausibility, multiplied by −1) values in each experimental condition for the outcome variable (N400) and for each of our predictors. Note that the human derived metrics of cloze probability and plausibility reflect our experimental design. The Best Completions were intended to be predictable, while the Related, Unrelated, and Implausible conditions were designed to be unexpected, with Related and Unrelated conditions equated for cloze probability. Similarly, Best Completions, Related, and Unrelated conditions were all intended to be plausible, whereas the Implausible condition was intended to be implausible. Figure 1 indicates that all of the computational metrics were associated with differences between Best Completions and Implausible endings. Related and Unrelated conditions were quite similar on some metrics—such as GloVe contextual cosine similarity (GloVe CCS) and fastText CCS—and differed on others, such as GPT-3 surprisal and both measures of BCCS.

**Figure 3.**
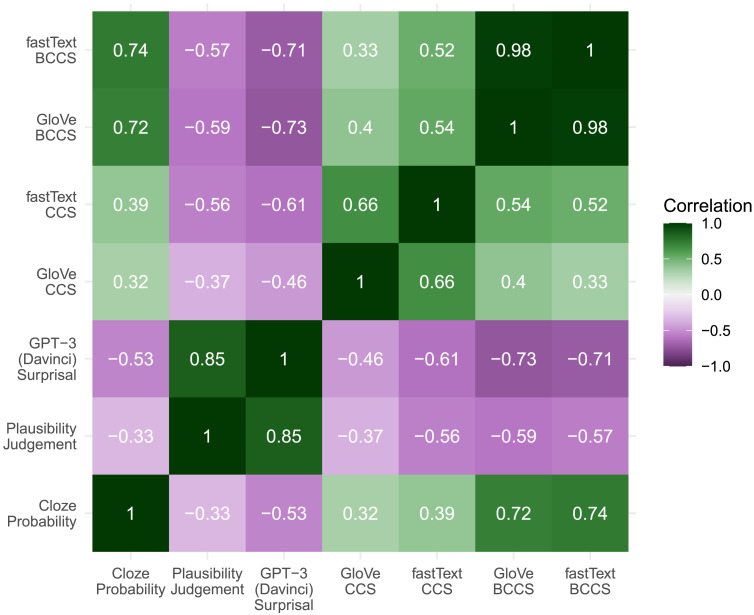
Heatmap of correlations between predictors.

Figure 3 presents a heatmap of correlations between the various predictors used in the regression analyses below. Recall that contextual cosine similartity is the cosine of the angle between the vector for each word and the mean of the vectors for each of the words in the preceding sentence context and serves as an operationalization of contextual semantic similarity. Best completion cosine similarity is the cosine of the angle between the vector for each word and the vector for the word that is the best completion for the sentence frame and is relevant for some multiple systems accounts. Although the two kinds of embeddings (GloVe and fastText) yielded virtually identical estimations of similarity between pairs of words—as reflected in the 0.98 correlation between GloVe BCCS and fastText BCCS—they differed somewhat in their estimates of contextual semantic similarity as GloVe CCS and fastText CCS had a correlation coefficient of 0.66. Relative to GloVe CCS, fastText CCS was more associated with cloze probability (0.39 vs. 0.32), GPT-3 surprisal (−0.61 vs. −0.46), and plausibility (−0.56 vs. −0.37). Relative to GloVe CCS, the fastText CCS measure also showed more sensitivity to the semantic relationship between each unexpected ending and the best completion, as evidenced by a greater correlation with fastText BCCS (0.52 vs. 0.33) and even with GloVe BCCS (0.54 vs. the 0.4 correlation between GloVe CCS and Glove BCCS).

GPT-3 exhibited similar correlations with cloze probability (−0.33) as did the contextual semantic similarity measures described above. Moreover, GPT-3 surprisals were highly correlated with human measures of plausibility (0.85), a level far greater than any of the other measures. As noted above, GPT-3 surprisal exhibited moderate negative correlations with both measures of CCS (−0.61 for fastText and −0.46 for GloVe). GPT-3 exhibited even higher correlations with the measures of BCCS (−0.71 for fastText and −0.73 for GloVe), presumably due to the way BCCS implicitly incorporates the predictions of the best completion.

### Single Factor Accounts

To begin our investigation, we evaluate how well each metric predicts N400 amplitude, allowing us to validate our statistically derived metrics (surprisal and cosine similarity) against the more traditional human-derived metrics (cloze probability and plausibility judgements) and to directly compare the former in their ability to predict N400 amplitude.

In order to compare these predictors, we constructed linear mixed-effects regression models with each variable of interest as a fixed effect and used Akaike’s information criterion (AIC; Akaike, 1973) to compare the regressions’ fits of the neural data. Each regression had a fixed effect of either cloze probability, plausibility judgement, GloVe Contextual Cosine Similarity, fastText Contextual Cosine Similarity, GPT-3 surprisal, and experimental condition. Note that we use cloze probability rather than cloze surprisal (i.e., log-transformed cloze probability) because previous work has not shown any clear evidence that the latter is a better predictor of N400 amplitude (see Michaelov et al., 2023; Szewczyk & Federmeier, 2022). In addition, one experimental condition (Implausible) was entirely made up of stimuli where critical words had a cloze probability of zero, which cannot be log-transformed, and “smoothing” such zero values to allow log-transformation by assigning them a very low probability also introduces problems for analysis (Nieuwland et al., 2021).

Because the inclusion of random slopes often leads to problems with convergence and singular fits, we chose to utilize a parsimonious random effects structure (Bates et al., 2018) in our regressions. Consequently, model comparison always involves regression models with the same random effects structures, which allows for comparison across models with different predictors. All regressions had random intercepts of sentence frame, subject, and electrode, as well as fixed effects of word frequency (calculated using the *wordfreq* Python package; Speer et al., 2018) and orthographic neighborhood size as operationalized by Coltheart’s *N* (Coltheart et al., 1977; calculated using MCWord; Medler & Binder, 2005). We also included a random intercept for each critical word because critical words often occurred in more than one condition.

**Figure 4.**
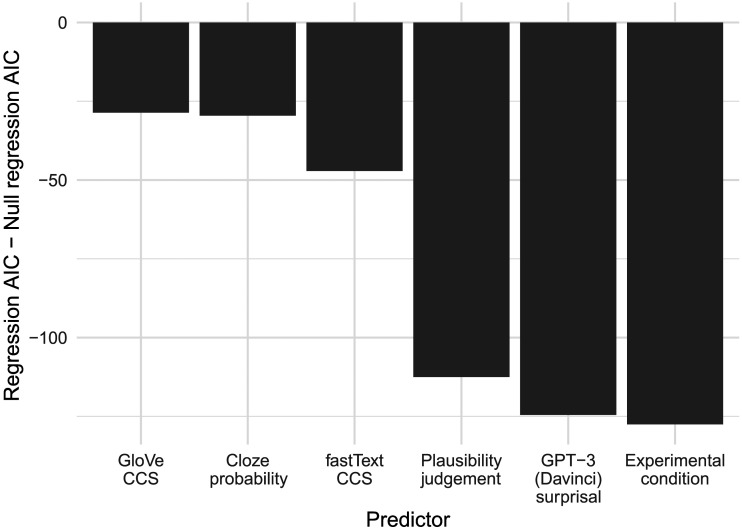
The AICs of the regressions resulting from the single factor analyses. AIC = Akaike’s information criterion.

The AIC of each regression, normalized by the AIC of the null regression (which includes the same random effects structure as the other regressions, and only word frequency and orthographic neighborhood size as fixed effects) is presented in Figure 4.

Of the continuous predictors, Figure 4 indicates that the best-fitting regression is that including GPT-3 surprisal as a main effect, suggesting GPT-3 surprisal is the best predictor of N400 amplitude. GPT-3 surprisal is followed by human plausibility judgements, which are followed by fastText CCS, which in turn is followed by cloze probability and GloVe CCS. It is generally accepted that a difference in AIC of 4 indicates a substantial difference (Burnham & Anderson, 2004), and the difference between cloze probability and GloVe CCS is only 0.9; thus it is not clear from our analysis which is the better predictor.

Figure 4 also indicates that the regression including experimental condition (a categorical variable with four levels: Best Completion, Related, Unrelated, and Implausible) has a lower AIC than the GPT-3 surprisal regression. However, experimental condition should not be considered to reflect a single variable in the way that the other individual predictors do because it includes information about predictability, plausibility, and RBC. Additionally, the experimental condition regression has an AIC of only 3 less than the GPT-3 surprisal regression; thus it is not clear that experimental condition is in fact a better predictor than GPT-3 surprisal.

We also ran likelihood ratio tests on each of the predictors listed in Figure 4, comparing each regression to a null regression, that is, one without the predictor of interest but all other fixed and random effects. All variables were significant predictors of N400 amplitude (GloVe CCS: *χ*^2^(1) = 30.6, *p* < 0.001; Cloze: *χ*^2^(1) = 31.6, *p* < 0.001; fastText CCS: *χ*^2^(1) = 49.1, *p* < 0.001; Plausibility: *χ*^2^(1) = 114.5, *p* < 0.001; GPT-3 Surprisal: *χ*^2^(1) = 126.6, *p* < 0.001; Condition: *χ*^2^(3) = 133.6, *p* < 0.001).

### Combined Accounts

**Figure 5.**
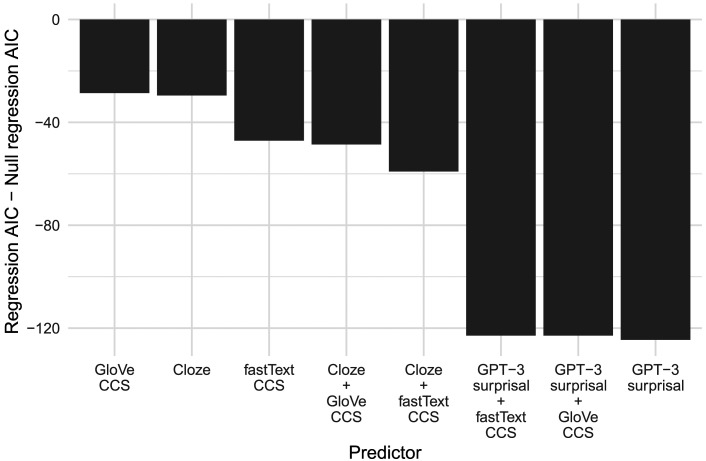
The AICs of the regressions resulting from the two-variable analyses corresponding to combined accounts.

The GPT-3 surprisal metric was chosen to model a prediction-based account of the N400, and GloVe CCS and fastText CCS were chosen to model the contextual semantic similarity accounts. As noted above, some authors have suggested the N400 indexes neurocognitive systems sensitive both to the predictability of a word and to its similarity to the semantic context. To investigate the viability of such combined accounts, we compare the AICs of regressions including a single variable corresponding to either prediction or contextual semantic similarity, with the AICs of regressions also including one of the other. Thus, we look at all combinations of prediction (viz., cloze probability and GPT-3 surprisal) with CCS metrics. The results are presented in Figure 5. A comparison of the AICs suggests that cloze probability and the two CCS metrics explain variance in N400 amplitude not explained by the other. This is borne out by the likelihood ratio tests: After correcting for multiple comparisons the cloze probability regression is improved by adding either GloVe CCS (*χ*^2^(1) = 21.0, *p* < 0.001) or fastText CCS (*χ*^2^(1) = 31.6, *p* < 0.001) as a predictor; and conversely, the GloVe (*χ*^2^(1) = 22.0, *p* < 0.001) and fastText (*χ*^2^(1) = 14.0, *p* < 0.001) regressions are each improved by adding cloze probability as a predictor. This suggests cloze probability and the CCS metrics explain non-overlapping portions of the variance in N400 amplitude. However, the same is not true of GPT-3 surprisal—while adding GPT-3 surprisal improves both the GloVe (*χ*^2^(1) = 96.3, *p* < 0.001) and fastText (*χ*^2^(1) = 77.8, *p* < 0.001) CCS regressions, the GPT-3 surprisal regression is not improved by adding either GloVe CCS (*χ*^2^(1) = 0.4, *p* = 1.000) or fastText CCS (*χ*^2^(1) = 0.4, *p* = 1.000). Thus GPT-3 explains variance left unexplained by the CCS measures, while the information provided by CCS was largely redundant with that provided by GPT-3.

### The Plausibility Effect

To test how well our metrics explain the variance in N400 amplitude traditionally explained by plausibility judgements, here we investigate whether the addition of plausibility as a predictor improves the GPT-3 surprisal regression, the cloze + GloVe CCS regression, and the cloze + fastText CCS regression. These regressions were selected because they were the models including each of our original three statistically derived metrics (that is, for predictability and for contextual semantic similarity) that performed the best in accounting for observed variance in N400 amplitude. Of these, we can consider the GPT-3 surprisal regressions as relevant to the predictive preactivation account of the N400 and the cloze + CCS regressions as relevant to multiple systems accounts.

**Figure 6.**
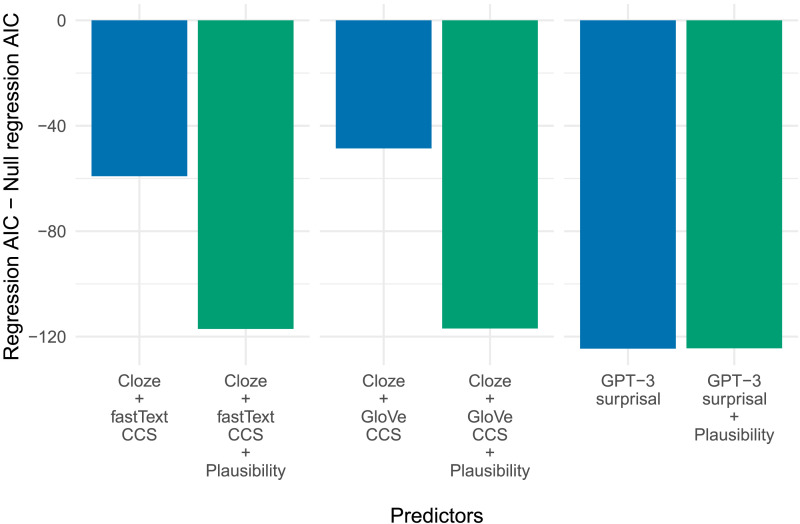
The AICs of the regressions resulting from the analyses investigating whether the single-factor and combined models account for the effect of plausibility.

Shown in Figure 6, the results indicate that even when combined with cloze probability (and thus, when part of a combined model that takes into account predictability as well as contextual similarity), the AICs of the regressions including GloVe (*χ*^2^(1) = 70.3, *p* < 0.001) and fastText (*χ*^2^(1) = 60.0, *p* < 0.001) CCS are improved by the addition of plausibility as a predictor. By contrast, the GPT-3 surprisal regression is not improved by adding plausibility as a predictor (*χ*^2^(1) = 1.9, *p* = 0.715). Whereas neither CCS metric can model the N400 plausibility effect—even when combined with cloze—variance attributable to plausibility was captured by GPT-3 surprisal. Thus, predictability alone (operationalized by GPT-3 surprisal) can explain the apparent effect of plausibility on N400 amplitude.

### The Relatedness to the Best Completion Effect

Finally, we explore the extent to which RBC is captured by our three metrics. As with plausibility, we look at whether adding a metric of RBC improves regression fit, where we operationalize RBC as the cosine distance between the word embeddings of the best completion for each sentence frame and the critical word in each of the other conditions, that is, our BCCS metric. We used both GloVe and fastText to derive measures of BCCS.

**Figure 7.**
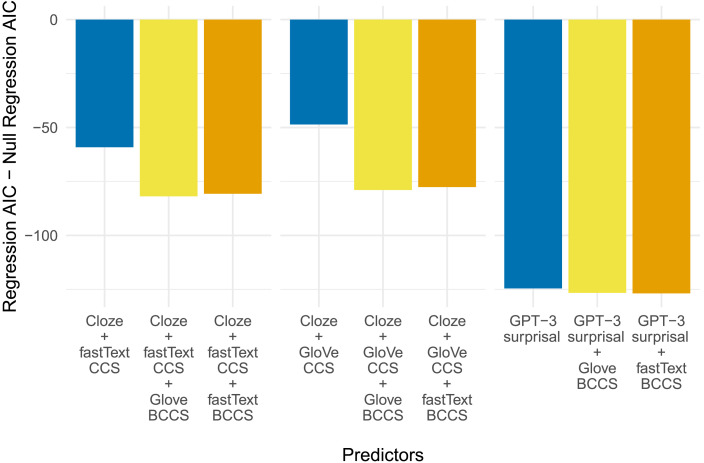
The AICs of the regressions resulting from the analyses investigating whether the single-factor and combined models account for the effect of the relatedness to the best completion.

As with plausibility, we investigate whether our previous best regressions for each of our three statistical metrics—that is, GPT-3 surprisal, cloze + GloVe CCS, and cloze + fastText CCS—are improved by the addition of BCCS to the model. The results are shown in Figure 7. The addition of GloVe BCCS to either cloze + CCS regressions led to improvements in model performance (Cloze + GloVe CCS: *χ*^2^(1) = 32.4, *p* < 0.001; Cloze + fastText CCS: *χ*^2^(1) = 24.8, *p* < 0.001); likewise the addition of fastText BCCS to either cloze + CCS regression led to significant improvements (Cloze + GloVe CCS: *χ*^2^(1) = 31.0, *p* < 0.001; Cloze + fastText CCS: *χ*^2^(1) = 23.6, *p* < 0.001). These results show that even when combined with cloze, contextual similarity cannot explain the RBC effect.

On the other hand, adding GloVe BCCS to the GPT-3 surprisal regression only reduces the AIC by 2, and adding fastText BCCS only reduces the AIC by 2.3; far from a clear improvement. When we run likelihood ratio tests, neither is found significantly to improve regression fit after controlling for multiple comparisons (GloVe BCCS: *χ*^2^(1) = 4.0, *p* = 0.192; fastText BCCS: *χ*^2^(1) = 4.3, *p* = 0.175). However, unlike all our other tests, this result is dependent on controlling for multiple comparisons—before this step, both BCCS metrics do appear to have a significant effect (GloVe BCCS: *p* = 0.044; fastText BCCS: *p* = 0.039). Thus, both when comparing AICs and when testing using likelihood ratio tests, while BCCS metrics may appear to improve model fit, they do not do so reliably.

One possible concern is that the extent to which the BCCS metrics predict N400 amplitude above and beyond surprisal may be undermined by the fact that for one condition (Best Completion), all BCCS values are, by definition, 1, as the critical word *is* the best completion. For this reason we also ran the same analysis excluding all data for Best Completions. The results were qualitatively the same: After correction for multiple comparisons, neither GloVe BCCS (*χ*^2^(1) = 5.0, *p* = 0.118; uncorrected *p* = 0.025) nor fastText BCCS (*χ*^2^(1) = 5.7, *p* = 0.087; uncorrected *p* = 0.017) significantly improved the regression already including GPT-3 surprisal.

## DISCUSSION

The aim of this article was to use current state-of-the-art language models to compare the predictions of two accounts of the neural activation underlying the N400 response—predictive preactivation versus contextual semantic similarity. To do this, we investigated how well GPT-3 surprisal—our best approximation of the kinds of predictions neurocognitive systems may make based on the statistics of language—predicts N400 amplitude. We compared this with the performance of GloVe and fastText contextual cosine similarity, our two best approximations of contextual semantic similarity based on the statistics of language. Finally, we compared this with the performance of combined models including both kinds of metrics. Based on this approach, we found that the predictive preactivation account explains more variance in N400 amplitude than the two models of contextual semantic similarity.

Below we consider the adequacy of predictive preactivation, contextual semantic similarity, and combined systems to account for the three kinds of N400 effects examined in the present study: expectancy effects, plausibility effects, and RBC. In each case, predictive preactivation provides a better account of N400 amplitude variation than does either a pure contextual similarity account or a multiple systems account. We end with a consideration of how the features of the deep learning language systems we used here relate to those of the language network in the brain.

### Expectancy Effects

While the close association between measures of contextual predictability and N400 amplitude is most naturally accounted for by the predictive preactivation account, advocates of contextual semantic similarity have argued that expectancy effects on the N400 arise because highly expected words share more semantic features with their context than do less expected words. This is demonstrated in computational modeling work by Ettinger et al. (2016), who uses the similarity between word2vec (Mikolov, Chen, et al., 2013; Mikolov, Sutskever, et al., 2013) representations of stimulus words and their contexts to account for the N400 amplitude differences between the best completions and their lower cloze counterparts in a widely cited study by Federmeier and Kutas (1999). Similarly, using wikipedia2vec embeddings (Yamada et al., 2020), Uchida et al. (2021) show that high cloze sentence continuations from a number of ERP language studies are more similar to their contexts than their less predictable counterparts.

In the present study, we likewise find that contextual similarity as measured both by GloVe CCS and fastText CCS is greater for best completions than for the other less expected endings. However, in a direct comparison of how well various measures of predictability versus contextual similarity account for variance in N400 amplitude, predictability as indexed by GPT-3 surprisal was the clear winner, providing a better account of the data than either GloVe CCS or fastText CCS. Moreover, the finding that regressions using both CCS measures improved when combined with cloze probability suggests these measures of contextual semantic similarity were unable to fully capture expectancy effects on the N400.

Of course, this same finding—that regressions with CCS measures are improved by the cloze probability factor—replicates work that supports the multiple systems account of the N400 (Federmeier, 2022; Lau et al., 2013). However, GPT-3 surprisal outperformed even these regressions (see Figure 5), suggesting that the predictive preactivation account of N400 is superior to both a pure contextual semantic similarity account and a combined systems account.

### Plausibility Effects

GPT-3 surprisal also accounts for more variance in N400 amplitude than our human-derived measure of cloze probability (in line with Michaelov et al., 2023), presumably due to its ability to capture subtle differences between highly unexpected items. Indeed, as Nieuwland et al. (2020) note, plausibility effects on the N400 might result because less plausible stimuli are also less predictable. Because cloze probability measures are limited in the extent to which they can adequately capture the predictability of highly improbable words, plausibility ratings may serve as a proxy for their predictability, allowing us to differentiate *very* low-probability completions from *extremely* low-probability ones. Of course, plausibility effects can also be accounted for in principle via contextual semantic similarity, since we would expect less plausible stimuli to be less related to their context.

Results of the present study, however, argue against the latter possibility as we find that even when combined with cloze probability, regressions including measures of contextual semantic similarity could not fully account for the plausibility effect. This finding serves as a conceptual replication of Nieuwland et al. (2020), who found that plausibility explains amplitude variance in the N400 not explained by either cloze probability or a contextual similarity metric derived from word2vec. However, unlike Nieuwland et al. (2020), we find that one metric of predictability—namely, GPT-3 surprisal—can successfully model the plausibility effect. In fact, it explains all the variance that plausibility judgements do. Thus, in contrast to the findings of Nieuwland et al. (2020), the results of the present study suggest that a single neurocognitive process—predictive preactivation—may be able to account for both predictability and plausibility effects on the N400. Whether this also applies to analyses across individual time steps within the N400 time window (of the kind carried out by Nieuwland et al., 2020) is a question for further research.

### Relatedness to Best Completion

As described in the Introduction, the RBC effect is not trivially explained by either predictability or contextual similarity; however, in principle it can be accommodated by either account, and there is some evidence for each. Under a predictability perspective, if semantic prediction is taking place, then we should expect words with a similar meaning to the best completion to be preactivated along with the best completion (DeLong et al., 2019). Consistent with this account, the predictions of computational language models have been used to successfully model the RBC effect (Michaelov & Bergen, 2020). Specifically, Michaelov and Bergen (2020) report that two language models (Gulordava et al., 2018; Jozefowicz et al., 2016) find related words to be more predictable than unrelated overall when modeling the stimuli from an experiment by Ito et al. (2016), and that one of these language models also shows the same pattern for stimuli from Kutas (1993).

According to the contextual semantic similarity account, the RBC effect results because words related to the best completion share semantic features with it. Thus, related words elicit reduced N400 for much the same reason the best completions do—their features have been preactivated because they are semantically related to the sentence context. This has also been successfully modeled computationally: Ettinger et al. (2016) find that the similarity between the word2vec (Mikolov, Chen, et al., 2013; Mikolov, Sutskever, et al., 2013) representation of a stimulus word and its preceding context demonstrates the RBC effect found by Federmeier and Kutas (1999)—words related to best completions were more semantically similar to the preceding context than were unrelated words.

The present study provides a conceptual replication of results reported both by Michaelov and Bergen (2020) and by Ettinger et al. (2016). Using GPT-3 surprisal we find that our Related completions were more predictable than the Unrelated ones (in line with Michaelov & Bergen, 2020); using fastText CCS we find that Related completions were more similar to the preceding context than were the Unrelated ones (in line with Ettinger et al., 2016). However, results in Figure 2—like those in both Michaelov and Bergen (2020) and Ettinger et al. (2016)—only demonstrate that overall, there is a significant difference in the predictability and in the contextual semantic similarity of Related and Unrelated completions as estimated by these computational language models; there is no direct comparison with human data.

The strength of the present study lies in our efforts to do just this. Direct comparison with the human N400 data suggests that the predictability metric from GPT-3 explains more variance in N400 amplitude than does either metric of semantic similarity to the context. Moreover, in our attempts to probe how well each metric captures the RBC effect, we utilized two computational measures of the semantic similarity between each best completion and the other three completions for the sentence frame: GloVe BCCS and fastText BCCS. As both the graphs in Figure 2 and the high correlation coefficient in Figure 3 suggest, the two BCCS measures were virtually identical with each other and both captured the human intuition that Related words were closer in meaning to the Best Completions than Unrelated words.

Regression models of N400 data indicate that the addition of either GloVe or fastText BCCS metrics to models already including cloze probability and GloVe or fastText CCS improves model fit (see Figure 7). This suggests that neither of our contextual semantic similarity metrics could fully account for the RBC effect—even when combined with cloze probability. On the other hand, the GPT-3 surprisal regression of N400 data was not substantially improved by the addition of either BCCS metric (see Figure 7), suggesting the variance associated with our measure of similarity to the best completion was largely redundant with that captured by GPT-3 surprisal. Moreover, the regression model including only GPT-3 surprisal outperformed all of the regression models with additive combinations of CCS, cloze probability, and BCCS. GPT-3 surprisal provides a better account of the RBC effect than does either a pure contextual semantic similarity account or a combination of prediction and contextual similarity.

While the superiority of GPT-3 over the contextual similarity measures is unambiguous, there is a bit of uncertainty regarding whether GPT-3 is improved by the addition of the BCCS metrics. In our statistical model comparisons, we do not consider regressions with a difference in AIC of less than 4 to differ meaningfully in their fit (following Burnham & Anderson, 2004). However, it is the case that numerically, the regressions including both GPT-3 surprisal and either GloVe or fastText BCCS have a lower AIC than that *only* including surprisal. Unfortunately, the outcome of the relevant likelihood ratio tests was also somewhat equivocal on this matter. After correcting for multiple comparisons, neither GloVe nor fastText BCCS explain a significant amount of the variance in N400 amplitude above and beyond what is explained by GPT-3 surprisal. Before correction, however, those comparisons were both significant at the 0.05 level. It is thus important to consider what might explain this (marginally) better fit to the data.

One straightforward explanation can be arrived at by further inspection of Figure 2. As can be seen, GPT-3 surprisal provides a good account of the difference in the expectancy between Best Completions and the Unrelated condition, and a good account of the difference between the Unrelated and the Implausible condition—impressions borne out by the analyses comparing surprisal to human-derived metrics of cloze probability and plausibility. The disconnect between GPT-3 surprisal and N400 data lies mainly in failing to fully capture the similarity in N400 amplitude between the Best Completions and the Related condition, as the latter elicit more positive N400 in humans than the GPT-3 regression model fits suggest. Thus, the addition of another variable that captures the difference between Related and Unrelated completions—variance not present in cloze probability or plausibility, and unreliable in the CCS metrics—may explain the improved fit with the addition of BCCS metrics. This may also explain the slightly lower AIC of the regression including the categorical variable of experimental condition in Figure 4.

Crucially, however, even if GPT-3 does not fully account for the RBC effect, the RBC effect observed here supports predictive preactivation as at least a partial account of the brain activity underlying the N400. If words semantically related to the best completion are facilitated in virtue of being related to the best completion, this presupposes the preactivation of information related to the best completion (DeLong & Kutas, 2020; see also Kuperberg et al., 2020). For example, it may be the case that the reason for the greater facilitation for related than unrelated words is that predictive processing involves the preactivation of conceptual semantic features rather than lexical items (Thornhill & Van Petten, 2012). Alternatively, it may be that there is a separate associative mechanism that activates words related to the best completion. In the first case, the preactivation of the related word occurs as part of a single predictive process; in the second, as a consequence. Both possibilities require the preactivation of the best completion—either the lexical item itself or its semantic features. Regardless, the present study clearly shows that, as operationalized here, predictive preactivation provides a better account of the RBC N400 effect than does contextual semantic similarity (see Figure 7).

Overall, in addition to being the best metric of predictability tested (in line with the results of Michaelov et al., 2023), GPT-3 surprisal also appears to successfully account for additional reported N400 effects, namely, that more plausible completions elicit smaller N400 responses than less plausible completions, and that words that are semantically related to the best (highest-cloze) completion elicit smaller N400 responses than unrelated words. In sum, with a good enough operationalization of contextual predictability, it is possible that we can reduce all effects observed during the temporal interval associated with the N400 to this single factor. The most parsimonious interpretation is that apparent effects of expectancy, plausibility, and RBC all index sensitivity to contextual predictability—and predictability derived from the statistics of language at that—suggesting N400 effects are due to a predictive preactivation process.

### Implications for Neural Mechanisms

Although we do not here treat any of the computational models used in this study as cognitive models, it is important to consider what the differences in the way that they work imply about that language network in the human brain. GPT-3 is a neural language model trained to optimize its estimates of the probability of upcoming words and how these values change with different amounts of linguistic context. Moreover, GPT-3 surprisal was the single best numerical predictor of N400 amplitude. On the other hand, GloVe and fastText, which model the relations between words, performed worse overall at predicting N400 amplitude. In this way, our results are highly compatible with predictive coding theories that suggest neural systems are constantly generating and updating an internal model of the environment (Allen & Tsakiris, 2018; Bendixen et al., 2012; Clark, 2013; Friston, 2010; Huang & Rao, 2011; McRae et al., 2019; Rao & Ballard, 1999; Shipp et al., 2013).

Applied to language, such approaches typically take the form of neural systems that generate predictions regarding upcoming words, using the word encountered at the next time step to generate a learning signal known as a prediction error (e.g., Elman, 1990). Indeed, something that we believe has been underappreciated in this regard is that the loss function used to train language models such as GPT-3, cross-entropy, is equivalent to surprisal (see Jurafsky & Martin, 2023, pp. 149–150). The close relationship we observed here between GPT-3 surprisal and N400 amplitude is perfectly in line with the suggestion that the N400 reflects a prediction-error based update of an internal language model (Bornkessel-Schlesewsky & Schlesewsky, 2019; Fitz & Chang, 2019; Hodapp & Rabovsky, 2021; Kuperberg, 2021; Kuperberg et al., 2020; Lewis & Bastiaansen, 2015; Rabovsky, 2020).

As Kuperberg et al. (2020) note, this account does not fit neatly into either retrieval (e.g., Brouwer et al., 2017; Brouwer & Hoeks, 2013; Kutas et al., 2006; Kutas & Federmeier, 2000; Lau et al., 2008; Van Berkum, 2009, 2010) or integration (e.g., Hagoort et al., 2009; van den Brink & Hagoort, 2004) accounts of the N400. Under our predictive coding account of the N400, the N400 is a measure of the neural activation elicited by a stimulus that was not already activated by prediction based on the preceding context. In this way, it indexes retrieval difficulty—the effort required to fully activate the neural representations needed to process the stimulus, which is reduced if some of these representations are already activated. By contrast, N400 amplitude could also be considered to index integration in that words that are easier to integrate with the preceding context are likely to be more strongly predicted (see, e.g., Kuperberg et al., 2020; Kuperberg & Jaeger, 2016). However, this only encompasses a limited subset of what could be considered integration difficulty—words that are highly anomalous, violate thematic roles, or lead to a substantial shift in the meaning of the preceding context instead appear to elicit later positivities (Coulson & Lovett, 2004; DeLong & Kutas, 2020; Kuperberg et al., 2020).

Our results are compatible in principle with a two-system account involving both contextual semantic similarity and predictive preactivation (as in Federmeier, 2022; Frank & Willems, 2017; Lau et al., 2013). However, given that the former does not explain any additional variance in the neural data, a predictive-preactivation-only account is more parsimonious. Further, in view of the correlation between GPT-3 surprisal and the CCS metrics (GloVe: *r* = −0.46; fastText: *r* = −0.61), it is possible that N400 effects previously explained as resulting from contextual semantic similarity may be an artifact of its correlation with the contextual predictability of words. Indeed, direct evidence of a neurocognitive process implementing contextual semantic similarity-based activation would require demonstrating an effect of contextual semantic similarity that cannot be linked to its contextual predictability.

One possible candidate for an effect that would help to test this is the finding that in some contexts, highly anomalous words that violate thematic roles (A. Kim & Osterhout, 2005; Kuperberg et al., 2003; Nieuwland & Van Berkum, 2005) or temporal event structure (Delogu et al., 2019) do not elicit a larger N400 response than non-violating stimuli. For example, Kuperberg et al. (2003) find no significant difference in N400 amplitude between *For breakfast the eggs would only **eat*** and *For breakfast the boys would only **eat***, and Delogu et al. (2019) do not find a significant difference between *John entered the restaurant. Before long, he opened the **menu*** and *John left the restaurant. Before long, he opened the **menu***. In both cases, the critical word’s relation to the preceding context appears to nullify the increase in N400 amplitude one might expect from the degree of semantic anomaly. To the best of our knowledge, only one study (Michaelov & Bergen, 2020) has attempted to model this effect using the stimuli from A. Kim and Osterhout (2005), finding that the surprisal elicited by stimuli such as *The hearty meal was **devouring*** is significantly higher than that elicited by either *The hearty meal was **devoured*** or *The hungry boy was **devouring***, which differs from N400 amplitude where it is not significantly different from either. This would indeed suggest that predictability, and thus prediction, cannot fully account for the N400 effect. However, it is important to note that this study used recurrent neural networks, whose predictions have been found to correlate far less with N400 amplitude than contemporary transformer language models (Merkx & Frank, 2021; Michaelov et al., 2023). Thus, whether this effect can be accounted for by contextual predictability alone is still an open question, and we believe a fruitful avenue for future research.

The results of using a language model to model the study carried out by A. Kim and Osterhout (2005) may also be valuable in better understanding the content of the preactivation underlying the N400 response. For example, a number of accounts argue that the preactivation underlying the N400 response is at the level of the semantic features of words (Federmeier, 2022; Kuperberg et al., 2020). While there is evidence that N400 amplitude is sensitive to phonological and grammatical features (DeLong et al., 2005; Fleur et al., 2020; Nicenboim et al., 2020; Otten et al., 2007; Urbach et al., 2020; Van Berkum et al., 2005), it may be that the shared semantic features between, for example, *devouring* and *devoured*, are sufficient to preactivate both words equally. Thus a semantically augmented language model may be able to better model the effect.

Alternatively, or in addition, it may be that the preactivation underlying the N400 operates at the sub-word level (as proposed by Smith & Levy, 2013) or specifically at the morphemic level, either in general or in cases where the redundant derived forms of words are not stored (for discussion, see Hanna & Pulvermüller, 2014). It may be that it is *devour* that is activated, and any additional activation conferred by *-ing* or *-er* suffixes is so subtle as to be undetectable in the scalp-recorded N400. This suggestion is in line with the finding that N400 amplitude is most sensitive to the predictability of content words (Frank et al., 2015). This could be investigated by testing language models with different tokenization schemes, for example, those where tokenization schemes are implemented that make tokens correspond more closely to morphemes (for discusion and attempts, see Bostrom & Durrett, 2020; Hofmann et al., 2021; Klein & Tsarfaty, 2020; Mohebbi et al., 2021; Yehezkel & Pinter, 2023).

Finally, it may be the case that surprisal measures derived from language models relate to aspects of the brain response to words in sentences beyond the N400. For example, some of the predictions of the recurrent neural networks tested by Michaelov and Bergen (2020) were better correlated with post-N400 positivities than the N400. The adequacy of different neural language models in fitting various aspects of the ERP waveform (such as those discussed in DeLong & Kutas, 2020; Kuperberg et al., 2020) is thus a promising area of further research and may help to shed light on language processing in the human brain.

A further intriguing question is the role played by the statistics of language. GPT-3 is trained using only linguistic data, meaning its predictions are solely based on the statistical patterns available in their language input. By contrast, under the majority of contemporary accounts of the N400, world experience plays a key role in shaping the semantic representations that are activated during language comprehension (e.g., Amsel et al., 2015; Chwilla & Kolk, 2005; Federmeier, 2022; Hagoort et al., 2004; Kutas & Federmeier, 2011; Metusalem et al., 2012; Paczynski & Kuperberg, 2012). For this reason, it may be surprising that a model deriving its semantics solely from language is able to predict words in a way that so closely appears to match the activation of words in humans. One possible conclusion to draw from this is that humans, too, base their linguistic predictions on the statistics of language.

While there is evidence that both humans (Bedny et al., 2019; J. S. Kim et al., 2021; Marmor, 1978; Saysani et al., 2018) and language models (Abdou et al., 2021; Li et al., 2021; Piantadosi & Hill, 2022) can learn a wide range of semantic information based on language input alone, language models have also been found to have limitations. Specifically, language models trained only on language data struggle to learn perceptual properties of entities (Forbes et al., 2019) and are limited in the kinds of novel affordances they can infer for objects (Jones et al., 2022). By contrast, N400 amplitude is sensitive to people’s understanding of the sensorimotor properties of the referents of words (Amsel et al., 2013, 2014, 2015; Wu & Coulson, 2011). Perhaps most importantly, language alone drives the probability estimates of GPT-3, whereas the N400 is sensitive to the contextual congruity of faces, gestures, images, environmental sounds, and action sequences (see Kutas & Federmeier, 2011, for review). Further work is needed to determine how other, non-linguistic sources of information influence the N400 response.

## ACKNOWLEDGMENTS

For their helpful comments and valuable discussion, we would like to thank the anonymous reviewers, the attendees of the 26th Architectures and Mechanisms for Language Processing Conference and the 43rd Annual Meeting of the Cognitive Science Society, as well as the Language and Cognition Laboratory, Brain and Cognition Laboratory, and Kutas Cognitive Electrophysiology Laboratory members and lab meeting attendees. We would also like to thank the San Diego Social Sciences Computing Facility Team for their technical assistance. The RTX A6000 used for this research was donated by the NVIDIA Corporation.

## FUNDING INFORMATION

James A. Michaelov, Center for Academic Research and Training in Anthropogeny (https://dx.doi.org/10.13039/100014515), CARTA Merle‐Smith Fellowship Award.

## AUTHOR CONTRIBUTIONS

**James A. Michaelov**: Conceptualization: Lead; Data curation: Lead; Formal analysis: Lead; Investigation: Equal; Software: Lead; Visualization: Lead; Writing – original draft: Equal; Writing – review & editing: Equal. **Megan D. Bardolph**: Conceptualization: Supporting; Data curation: Equal; Investigation: Equal; Software: Supporting; Writing – review & editing: Supporting. **Cyma K. Van Petten**: Resources: Supporting; Supervision: Supporting; Writing – review & editing: Supporting. **Benjamin K. Bergen**: Resources: Supporting; Supervision: Supporting; Writing – review & editing: Supporting. **Seana Coulson**: Conceptualization: Supporting; Resources: Lead; Supervision: Lead; Writing – original draft: Equal; Writing – review & editing: Equal.

## DATA AND CODE AVAILABILITY STATEMENT

The data, code, and analysis scripts used for the present study are available at https://osf.io/pysbc.

## TECHNICAL TERMS

Event-related brain potential (ERP): Electrical activity in the brain recorded at the scalp using electroencephalography, time-locked to a particular category of stimulus or response event.
N400: A negative-going component of the event-related brain potential that peaks roughly 400 ms after stimulus presentation; thought to indicate processing.
Predictive coding: A theory under which the functional role of neural systems centers on prediction and learning from prediction error.
Language model: A computational system that calculates the contextual probability of a word. Most contemporary language models are transformer neural networks.
Transformer neural network: A state-of-the-art neural network architecture that uses an “attention” mechanism that allows it to make use of a long context when making predictions.
Surprisal: The negative logarithm of a probability (in this study, the probability of a word in context).
